# Psychopathic Personality Works Better than CU Traits for Predicting Fearlessness and ADHD Symptoms among Children with Conduct Problems

**DOI:** 10.1007/s10862-018-9651-0

**Published:** 2018-03-09

**Authors:** Louise Frogner, Anna-Karin Andershed, Henrik Andershed

**Affiliations:** Center for Criminological and Psychosocial Research (CAPS), School of Law, Pscyhology and Social Work, Örebro University, SE-70182 Örebro, Sweden

**Keywords:** Attention-deficit hyperactivity disorder, Callous-unemotional traits, Conduct problems, Early childhood, Fearlessness, Psychopathic traits

## Abstract

Children with early-onset conduct problems (CP) are at great risk for future behavior problems, and this risk seems to increase when CP co-occur with psychopathic traits. Even though studies are indicating that the entire psychopathic personality construct may be more useful in designating a meaningful subgroup of children with CP, research on psychopathic traits and CP in childhood have mainly focused on the role of callous unemotional (CU) traits. Prospective longitudinal data of 1867 3- to 5-year-olds (47% girls) followed annually for two years was used to compare groups of children with different combinations of CP and psychopathic traits on fearlessness and Attention-Deficit Hyperactivity Disorder (ADHD) symptoms. Children with CP and psychopathic personality had higher baseline and stable levels of ADHD symptoms than children with CP only or children with CP and concurrent CU traits, while baseline levels of fearlessness did not differ. They were also more likely to display stable levels of the risky combination of CP and ADHD symptoms. Results were similar for boys and girls. Findings indicate that there are reasons to consider other traits and behaviors as specifiers for subgroups of children with CP over and above CU traits, in order to optimize both diagnostic practice and treatment outcomes.

Children with conduct problems (CP) constitute a heterogeneous group, with different variations and combinations of risk and protective factors for the development of future behavior problems, such as antisocial or criminal behavior. Based on this notion, CU traits, such as lack of remorse, guilt and empathy, and shallow emotions, have been included in the Diagnostic and Statistical Manual of Mental Disorders (DSM-5; American Psychiatric Association 2013) as a Limited Prosocial Emotions (LPE) specifier for the Conduct Disorder (CD) diagnosis (see Frick and Moffitt 2010). Previous research has mainly focused on comparing children with CP and CU traits to children with CP without concurrent CU traits, and not controlling for other empirically based dimensions of psychopathic traits, i.e., impulse control and narcissism (Frick et al. 2014). However, as the full psychopathic personality seems to be related to CP among children in a similar way as among adolescents and adults, i.e., elevated levels of several dimensions of psychopathic traits increase the risk for antisocial behavior, criminality and recidivism (e.g., Colins et al. 2015; Kotler and McMahon 2005; Moffitt et al. 2002), it has been suggested that dimensions of psychopathic traits other than CU traits (i.e., the affective dimension) should be considered in relation to the development of CP. Similar to among adolescents and adults (e.g., Andershed et al. 2008; Cooke and Michie 2001), studies repeatedly have shown that child psychopathic personality consists of at least three dimensions of traits and behaviors (Colins et al. 2014; Colins et al. 2016a, b; Frick et al. 2000; Jones et al. 2006). This suggests that by looking at children with CP and concurrent CU traits only, we are potentially overlooking important information about what contributes to maintaining the behavior problems (see also Frogner et al. 2016). Consequently, more research is needed that investigates the potential usefulness of using other psychopathic traits than CU traits only in understanding severe CP. The aim of the present study was to compare groups of children with CP only, children with a combination of CP and CU traits, and children displaying CP and the full psychopathic personality, i.e., three dimensions of psychopathic traits (Colins et al. 2014; Cooke and Michie 2001) on traits and behaviors that in research have shown to be important for the development of CP, in this case, fearlessness and Attention-Deficit Hyperactivity Disorder (ADHD) symptoms.

## Conduct Problems and Psychopathic Traits

Psychopathic personality, as used with adults, is commonly comprised by three dimensions of traits and behaviors, i.e., an interpersonal, an affective, and a behavioral/lifestyle dimension (see Cooke and Michie 2001), and while often stating this, researchers investigating psychopathic personality in children still focus mainly on CU traits only (see Frick et al. 2014 for a review). There are numerous studies showing the increased risk for serious and persistent behavior problems for children with concurrent CP and CU traits, but these studies have often focused on comparing children with the combination of CP and CU traits to children with CP only, without controlling for other dimensions of psychopathic traits. Not controlling for other psychopathic traits when examining children with elevated levels of CU traits means that there is no way of knowing whether the association between CU traits and CP is confounded by other co-occurring dimensions of psychopathic traits. Furthermore, studies have shown that the predictive ability of CU traits for CP is reduced when controlling for other psychopathic traits (Barry et al. 2000; Corrado et al. 2004; Frick et al. 2000). This is important from the perspective that it may be other and/or additional drivers contributing to the development of CP that need to be in focus in interventions. In addition, previous research has demonstrated the importance of including other dimensions of psychopathic traits in relation to CP when extending the psychopathic personality to childhood (Colins et al. 2014; Frick et al. 2000; Salekin and Lynam 2010). In line with this reasoning, a recent study of preschool children (the same cohort as in the present study) tested the predictive ability of early-onset CP and concurrent psychopathic traits on stable levels of CP over time, focusing on the comparison between children with CP and concurrent CU traits, and children with CP and concurrent psychopathic personality (Frogner et al. 2016). The results showed that the combination of CP and psychopathic personality (i.e., high levels on all three dimensions of psychopathic traits) was a stronger predictor of stable CP than the combination of CP and CU traits for boys, and an equally strong predictor for girls. This indicates that psychopathic traits other than CU, together with early-onset CP, play an important role in the development of severe and persistent behavior problems, and that including other psychopathic traits and not only CU traits may be a better approach for identifying an especially problematic subgroup of children with CP.

## Fearlessness and ADHD Symptoms as Risk Factors for CP

Children who develop CP often display multiple risk factors, where the sheer amount of risk factors means increased risk in itself, i.e., a cumulative risk model is highly adequate to understand the development of CP (Appleyard et al. 2005; Calkins et al. 2007). This means that children with CP often will display multiple problematic traits and behaviors other than CP in itself, that increase risk for future CP and that most likely are possible to identify and target with interventions (Loeber and Farrington 2000; Moffitt and Caspi 2001). Two theoretically and empirically relevant risk factors for severe and stable forms of CP, with substantiated predictive ability for future behavior problems, are fearlessness and ADHD symptoms (Caspi 2000; Frick et al. 1999; Moffitt 1993; Shaw et al. 2003; 2005). Also, both fearlessness and ADHD symptoms encompass traits and behaviors that are informative for early childhood interventions, especially in children with concurrent CP (e.g., Abikoff and Klein 1992; Hawes et al. 2014; Kolko and Pardini 2010). Research has shown that early childhood CP and ADHD symptoms, both separately and in combination, predict CP for males, but not for females, and that impulsivity seems to be more stable among boys with CP (Babinski et al. 1999; Berkout et al. 2011; Colder et al. 2002; Côté et al. 2002). Thus, it is important, with regard to amount of risk, severity, and treatment needs, to study boys and girls with CP separately.

### Fearlessness

Children with high levels of fearlessness often seek out novel situations to test limits, and usually do not fear negative consequences of misbehavior, i.e., they are not as sensitive to punishment as other children, which in turn affects their possibilities to socialize and adapt to prosocial norms (Dadds and Salmon 2003; Frick and Sheffield Morris 2004; Shaw et al. 2003). Studies have shown that fearless children are more likely to exhibit early-onset CP, as well as severe and stable forms of CP, than other children (e.g., Calkins et al. 2007; Moffitt 1993; Shaw et al. 2003; 2005).

### ADHD

ADHD commonly appears in early childhood, persists over time, and is often comorbid with ODD and CD (Biederman 2005). Research has repeatedly linked ADHD in childhood to the development of both severe and persistent CP, such as antisocial and criminal behavior, among both boys and girls (Caspi 2000; Fletcher and Wolfe 2009; Lee and Hinshaw 2006; Loeber and Farrington 2000; Mannuzza et al. 2008; Moffitt 1993; Murray et al. 2010; Sibley et al. 2014). Also, according to some research both boys and girls with ADHD in combination with CP, between preschool and the age of thirteen, seem to be at greater risk for future violent, antisocial, and criminal behavior than individuals displaying ADHD symptoms only (Mordre et al. 2011; Satterfield et al. 2007; Simonoff et al. 2004; Stormont 2000). Many of the children with stable CP also seem to follow stable trajectories of ADHD symptoms, indicating that these traits and behaviors follow similar developmental paths (López-Romero et al. 2015; Nagin and Tremblay 2001; Shaw et al. 2005).

### Fearlessness, ADHD Symptoms, and Psychopathic Traits

Both fearlessness and ADHD symptoms are associated with psychopathic traits. Fearlessness has been linked to children’s levels of CU traits (e.g., Barry et al. 2000; Fanti et al. 2015; Frick and Sheffield Morris 2004). However, the occurrence of CU traits seems to be closely linked to co-occurrence of other psychopathic traits, and, in turn, all these traits are related to fearlessness. A longitudinal study of a large preschool sample (the same cohort as in the present study) showed that children with stable high levels of CP and CU traits in combination also displayed stable high levels of fearlessness as well as high levels of other psychopathic traits (Klingzell et al. 2016). Research has also shown that high levels of psychopathic traits, and not only CU traits, mediate the relation between fearlessness and offending in young adulthood (Kubak and Salekin 2009). One study showed positive zero order correlations between fearlessness and ADHD symptoms, and the three separate psychopathic traits dimensions (Colins et al. 2014). However, when controlling for the other two dimensions of psychopathic traits correlations became close to zero, indicating that all dimensions of psychopathic traits matter in the correlation between each separate dimension, and fearlessness and ADHD symptoms. Also, levels of fearfulness at age three seem to be associated with high ratings on psychopathy traits, over and above CU, both among both boys and girls (Glenn et al. 2007). Boys with chronically high levels of CP are commonly characterized by high levels of fearlessness (Shaw et al. 2003). Low fearfulness has also been shown to predict CP among girls, whereas low fearfulness only predicted CP in interaction with time and high activity levels among boys (Colder et al. 2002). In conclusion, level of fear seems to be a crucial risk factor for severe and persistent CP among both boys and girls.

The combination of ADHD symptoms and CP also seems to be associated with psychopathic traits, and not only with CU traits. For example, a study showed that boys and girls between the ages of 10 and 18 with high ratings of psychopathic personality also displayed the highest levels of both CD and ADHD (both symptoms and diagnoses) compared to children with high levels of only one single dimension of psychopathy, i.e., CU, impulsivity, or narcissism (DeLisi et al. 2014; Frick et al. 2000). It is, however, important to note that there is an overlap between the behavioral (impulsivity) factor of the psychopathic personality (or the psychopathy syndrome) and the DSM ADHD hyperactivity/impulsivity type as both scales assess impulsivity and sensation seeking. This overlap is likely to carry a bulk of the association between the psychopathic personality and ADHD symptoms.

## Aims and Hypotheses

Based on the existing knowledge regarding CP and related risk factors, and psychopathic traits, it seems both relevant and possible to identify more homogenous subgroups of children with CP, in order to improve diagnostic practice as well as interventions to prevent and treat the development of severe CP. We question whether the combination of CP and CU traits, i.e., the DSM-5 LPE specifier for CD, is the only, or even the best way, to identify a more homogeneous group of CP children at risk. Therefore, the more specific aim of the present study was to examine subgroups of children with CP by comparing them on levels of other known risk factors for CP, in this case fearlessness and ADHD symptoms, both cross-sectionally and longitudinally. The main subgroups contrasted against each other in the present study are children with high levels of CP only, children with high levels of CP and CU traits, and children with high levels of CP and all three dimensions of the full psychopathic personality (i.e., high levels of the affective, interpersonal and behavioral dimensions of traits and behaviors).

Our hypotheses were that children with high levels of CP and the full psychopathic personality would display higher levels of both baseline and stable fearlessness and ADHD symptoms compared to both children with high CP only, and more importantly, compared to children with the combination of high CP and high CU. And even more importantly, we hypothesized that displaying CP and psychopathic personality would increase the risk for stable fearlessness and ADHD symptoms over a two-year period, compared to when exhibiting CP and CU traits. These precise comparisons are almost non-existent in previous research, but there are clear indications that the combination of CP and psychopathic personality implicates a great risk for developing CP (Colins et al. 2014; Frick et al. 2000; Frogner et al. 2016). Finally, because the combination of CP and ADHD symptoms is associated with risk for long-lasting criminality (Lynam 1996; Manuzza et al. 2004; Waschbusch 2002) we also wanted to examine whether children with high levels of CP and psychopathic personality would be more likely to display the combination of stable CP and ADHD symptoms over time, compared both to children with high CP only, and children with the combination of high CP and high CU. We also include children with CP but no psychopathic traits, children with CU traits but no CP, and children with psychopathic personality but no CP in the analyses for exploratory purposes. The analyses were conducted for boys and girls separately in order to explore potential differences in patterns across gender.

## Method

### The SOFIA Study

Data from the SOFIA study (*Social and Physical Development, Interventions and Adaptation*) were used in this study. The SOFIA study is an ongoing prospective longitudinal study, designed to provide a better understanding of the correlates and causes of heterogeneity in children’s behavior, social adjustment, and psychological and physical health. The normal population cohort targeted in the SOFIA study resides in a mid-sized Swedish municipality with approximately 85,000 citizens. In terms of proportion, the municipality is similar to the rest of Sweden with regard to age, sex, education level, level of employment, and the mixture between urban and rural areas. Active consent for participation was solicited from parents of 2121 children (83.4% of the initial target population).

In the present study, data from the first three waves of the SOFIA study, collected in 2010 (Baseline), 2011 (1-year follow-up), and 2012 (2-year follow-up) were analyzed. Data were collected between March and September each year, and exclusively through questionnaires answered by preschool teachers. The questionnaires consisted mainly of multiple-choice questions regarding the children’s behavior, their psychological and social wellbeing and adjustment, physical and medical status, as well as perceived need of, and given support and interventions for the children during the past six months.

### Participants

At baseline, the mean age of the participating children was 3.86 years (*SD* = 0.86), with 34.4% of children being 3-year-olds, 33.1% 4-year-olds, and 32.5% 5-year-olds.^1^ Responses for 2113 (99.6%) of the participating children were received from preschool teachers. At the 1-year follow-up, when the children were 4–6 years old, ratings for 2041 (96.2%) of the original participating children were received from teachers, and at the 2-year follow-up, when the children were 5–7 years old, ratings for 1934 (91.2%) children were received. This study only included children with valid information on study variables, which rendered a sample of 1867 (88%; 47% girls).

#### Non-Participants

To conclude whether the non-participating group differed on important dimensions from the participating group, 30 randomly chosen parents were interviewed. The non-participating group did not differ significantly from the participating group concerning, for example, CP or internalizing problems of the children, socio-economic status of the parents, or the country of origin of the father and the child.

### Measures

The included measures of Conduct Problems, Psychopathic Traits, Fearlessness, and ADHD symptoms have all been applied in previous studies, showing good internal consistency and external validity (Colins et al. 2014; Colins et al. 2016a, 2016b; Frogner et al. 2016; Klingzell et al. 2016; Somma et al. 2015). All measures were framed to assess the child’s behavior for the past six months.

#### Conduct Problems

CP was assessed by teachers’ ratings of 10 items of ODD and CD symptoms from the DSM IV-TR (American Psychiatric Association 2000) on a 5-point Likert Scale (1 *= Never,* 2 *= Rarely,* 3 *= Sometimes,* 4 *= Often,* 5 *= Very often*). Examples of items are “Has been very angry”, “Has violated important rules in preschool/school”, and “Has teased others”. Cronbach’s alpha for boys and girls respectively was α = .93, and α = .92 at baseline, α = .95, and α = .93 at the 1-year follow-up, and α = .95, and α = .95 at the 2-year follow-up.

#### Psychopathic Traits

The three dimensions of psychopathic traits were assessed using the CPTI (Child Problematic Traits Inventory; Colins et al. 2014) with teacher ratings. The CPTI consists of 28 items and was administered to the respondents as a part of the questionnaire; for this study only baseline ratings were used. Specifically, 8 items were used to measure the Grandiose-Deceitful dimension (GD), and 10 items each for the Callous-Unemotional (CU) and Impulsivity, Need for Stimulation (INS) dimensions. The response scale for each item ranges from 1 = *Does not apply at all*; 2 = *Does not apply well*; 3 = *Applies fairly well*; to 4 *= Applies very well*. Examples of items are “Often lies to get what he/she wants”, and “Is often superior and arrogant towards others” (GD; Cronbach’s alpha α = .92 for boys, α = .90 for girls), “Does not express guilt and remorse to the same extent as other children of the same age”, and “Seldom expresses sympathy for others” (CU; Cronbach’s alpha α = .95 for boys, α = .94 for girls), “Seems to have a great need for change and excitement”, and “Seems to do certain things just for the thrill of it” (INS; Cronbach’s alpha α = .93 for boys, α = .91 for girls). The factor structure, internal consistency and external validity of the CPTI has been tested in previous studies in different samples with good results (Colins et al. 2014; Colins et al. 2016a, b; Somma et al. 2015).

### Groups of Children with Conduct Problems, CU Traits, and Psychopathic Traits

The children were categorized by their levels of CP, and the three dimensions of psychopathic traits (i.e., GD, CU and INS) at baseline, in order to test the association between different combinations of CP and psychopathic traits, and subsequent fearlessness, ADHD symptoms, and combined CP and ADHD syptoms. To achieve this categorization, a cutoff of 0.5 *SD* within gender was used to dichotomize children into high (above cutoff) and low levels of CP, and the three dimensions of psychopathic personality. As boys commonly display CP to a larger extent than girls, and also display higher levels of CP during early childhood (see e.g., Moffitt and Caspi 2001), we made comparisons separately for boys and girls. Within gender cutoffs were deemed necessary in order to compare boys to boys and girls to girls, and to reduce the risk of overlooking possible gender specific patterns (e.g., Sprague and Verona 2010). Sensitivity analyses were also performed using stricter cutoffs which resulted in similar findings to those we report in the results section; these results are available upon request.

Using the 0.5 *SD* cutoffs, the categorizations rendered five mutually exclusive groups, that were used as predictors of future fearlessness, ADHD symptoms, and combined CP and ADHD symptoms in our regression analyses: *CP only* – elevated levels of CP but low on all three psychopathic dimensions (boys *n* = 29, girls *n* = 38), *CU only* – elevated levels of CU traits but low concurrent CP (boys *n* = 47, girls *n* = 32), *Psychopathic personality* (PP) *only* – elevated levels of all three psychopathic dimensions but low on concurrent CP (boys *n* = 25, girls *n* = 19), *CPCU* – elevated levels of CP *and* of concurrent CU traits, but low on the other two psychopathic dimensions (boys *n* = 14, girls *n* = 11), and *CPPP* – elevated levels of CP *and* all of the three psychopathic dimensions (boys *n* = 82, girls *n* = 76). For the regression analyses conducted, these five mutually exclusive groups were coded and entered as five separate dummy independent variables (0 = not fulfilling criteria for group membership; 1 = fulfilling criteria for group membership). Many children had low levels, i.e., below 0.5 *SD* within gender, of all included variables (*Low on all; n* = 499 boys and *n* = 486 girls). This Low on all group was included for the group comparison analyses, but not as a predictor in the regression analyses.

#### Fearlessness

The Child Fearlessness Scale (Colins et al. 2014; Colins et al. 2016a, 2016b; Klingzell et al. 2016) consists of six teacher rated items. Examples of items are: “He/she does not seem to be afraid for anything”, and “He/she never seems to get scared when someone is mad at him/her.” The response scale is: 1 = *Does not apply at all,* 2 = *Applies poorly,* 3 = *Applies fairly well,* and 4 = *Applies well.* The mean of the six items was calculated to gain the Fearlessness variable used in the present study. Cronbach’s alpha for boys and girls respectively was α = .91, and α = .87 at baseline, α = .91, and α = .90 at the 1-year follow-up, and α = .92, and α = .90 at the 2-year follow-up.

#### Attention-Deficit Hyperactivity Disorder (ADHD) Symptoms

This construct was assessed with teachers’ ratings of 18 items aimed to assess the diagnostic criteria of ADHD of the DSM-IV-TR (American Psychiatric Association 2000) using DuPaul’s ADHD scale (DuPaul et al. 1998). Examples of items are: “He/she is running around, clutching or climbing more than what is considered appropriate” and “He/she is inattentive on details or is careless.” The response scale is: 1 = *Never*, 2 = *Seldom*, 3 = *Sometimes*, 4 = *Often*, and 5 = *Very Often*. The items were framed to comprise the behavior of the child during the last six months. The mean of the 18 items was calculated to gain the ADHD symptoms variable used in the present study. Cronbach’s alpha was α = .96 for both boys and girls at baseline, α = .97, and α = .96 for boys and girls, respectively, at the 1-year follow-up, and α = .97 for both boys and girls at the 2-year follow-up.

#### Stability Measures

Measures of stable Fearlessness and stable ADHD symptoms were created as binary variables where children with mean values above 0.5 *SD* within gender (e.g., Sprague and Verona 2010) on both 1- and 2-year follow-up ratings were considered to have stable levels of these traits and behaviors (coded as 1), and children with 1- and 2-year follow-up ratings below this cutoff were considered not to have stable Fearlessness or ADHD symptoms, respectively (coded as 0). Also, a binary variable of high or low levels of combined ADHD symptoms and CP was created using the ratings of baseline ADHD symptoms, and 1- and 2-year follow-up ratings of ADHD symptoms and CP. Baseline CP was not included in the stable CP + ADHD symptoms variable in order to avoid multicollinearity issues, as baseline CP was used to create the predictor groups. Children with mean values above 0.5 *SD* within gender on all included variables were considered to have stable levels of CP + ADHD symptoms (coded as 1) and children with ratings below this cutoff were considered not to have stable CP + ADHD symptoms (coded as 0).

#### Parents’ SES and Origin

Parents’ socio-economic status (SES) was operationalized as parents’ mean level of education and income, using the z-transformed mean values. Parents’ origin was measured at baseline by asking whether the parents were born in Sweden or not.

### Statistical Analyses

Pearson correlations were estimated in order to examine the bivariate relationships among the study variables. All groups, i.e., CP only, CU only, PP only, CPCU, CPPP, and The group Low on all was then compared on levels of Fearlessness and ADHD symptoms at baseline through Analyses of Covariance (ANCOVAs), including the child’s age, and parents’ SES and origin as covariates. The groups were also compared on levels of stable Fearlessness and ADHD symptoms in contingency tables using *χ*^2^, as well as comparing column proportions, i.e., identifying differences in the number of children with high and low levels of Fearlessness and ADHD symptoms respectively between the five groups.

Logistic regressions were estimated to predict stable Fearlessness, stable ADHD symptoms, and stable CP + ADHD symptoms from early-onset CP and concurrent psychopathic traits. The five dummy coded groups (explained above), i.e., CP only, CU only, PP only, CPCU and CPPP, were entered as independent variables, together with covariates consisting of child’s age, parents’ SES, and parents’ origin. All analyses were conducted separately for boys and girls.

### Procedure

All data collection procedures used in the SOFIA study were approved by an ethics committee (#2009/429). For complete information about the recruiting and start-up procedure of the study, see Colins et al. (2014). An active consent form was filled out by the parents and collected by the preschool teachers, who then completed the questionnaires for children whose participation had been approved by parents. One teacher could answer the questionnaire for several children, as they responded concerning the children they were most familiar with at their preschool department. They also had the opportunity to choose if they wanted to respond via a secure web page or through a paper-and-pencil questionnaire. Preschool teachers answered the questionnaires, which took about 20 min to complete, as part of their daily work, and did not receive any personal compensation.

## Results

Pearson correlations between the child’s age, and CP, fearlessness, and ADHD symptoms ranged between -.10 and −.00 for boys, and between −.14 and .01 for girls, correlations between the parent’s origin, and CP, fearlessness, ADHD symptoms ranged between −.01 and .11 for boys, and between −.00 and .10 for girls, and correlations between the parent’s SES, and CP, fearlessness, and ADHD symptoms ranged between −.17 and −.08 for boys, and between −.13 and −.02 for girls. Age was not significantly correlated to levels of fearlessness at any point, neither among boys nor girls. SES was significantly and negatively related to all outcome variables among boys, but only to ADHD symptoms and 2-year follow-up CP among girls. Origin was significantly and positively related to 2-year follow-up ratings of CP and ADHD symptoms among both boys and girls, to baseline ADHD among girls only, and to 2-year follow-up fearlessness among boys, but not among girls.

Baseline CP was positively and significantly related to 1- and 2-year follow-up CP, as well as to baseline and 1- and 2-year follow-up fearlessness and ADHD symptoms, coefficients ranging between .36 and .70 for boys, and .25 and .65 for girls, see Table 1.

**Table 1:** Zero-order correlations between teacher rated conduct problems (CP), ADHD symptoms, and fearlessness at baseline, 1- and 2-year follow-up, with boys below, and girls above the diagonal

	CP	1-yr Follow-up CP	2-yr Follow-up CP	ADHD	1-year Follow-up ADHD	2-year Follow-up ADHD	Fearlessness	1-yr Follow-up Fearlessness	2-yr Follow-up Fearlessness
CP	–	.61***	.42***	.65***	.43***	.39***	.46***	.34***	.25***
1-yr Follow-up CP	.63***	–	.58***	.46***	.66***	.49***	.31***	.50***	.32***
2-yr Follow-up CP	.58***	.66***	–	.32***	.43***	.67***	.21***	.34***	.52***
ADHD	.70***	.47***	.41***	–	.61***	.49***	.53***	.39***	.23***
1-yr Follow-up ADHD	.50***	.69***	.49***	.61***	–	.60***	.33***	.56***	.31***
2-yr Follow-up ADHD	.45***	.51***	.70***	.49***	.60***	–	.30***	.39***	.50***
Fearlessness	.54***	.42***	.32***	.55***	.43***	.34***	–	.39***	.24***
1-yr Follow-up Fearlessness	.39***	.57***	.37***	.38***	.55***	.36***	.49***	–	.42***
2-yr Follow-up Fearlessness	.36***	.43***	.62***	.33***	.40***	.53***	.36***	.44***	–

*p < .05 **p < .01 ***p < .001

### Comparing Levels of Fearlessness and ADHD Symptoms

To test the hypothesis that children in the CPPP group displayed higher baseline and stable levels of fearlessness and ADHD symptoms than children in the CPCU group, we compared the groups through ANCOVA’s. Thus, the groups with different constellations of CP and psychopathic traits (presented in the Statistical Analyses section) were compared on levels of CP, psychopathic traits, and on both baseline and stable levels of fearlessness and ADHD symptoms, as well as on a combination of stable CP + ADHD symptoms. Results are displayed in Table 2, and show that both boys and girls in the CPPP group displayed significantly higher levels of baseline ADHD symptoms than the other groups. Neither CPPP boys, nor CPPP girls differed significantly from the respective CPCU groups in levels of baseline fearlessness. Also, none of the contingency tables rendered statistically significant differences between CPCU and CPPP children in stable levels of these traits and behaviors. However, as also can be seen in Table 2, between 34 and 49% of CPPP boys, and between 29 and 42% of CPPP girls, displayed stable levels of fearlessness, ADHD symptoms, and CP + ADHD symptoms, compared to only between 7 and 21% of CPCU boys, and between 0 and 9% of CPCU girls. This clearly indicates that children with CPPP are more likely to manifest these traits and behaviors consistently over time.

**Table 2:** Comparisons of groups with different combinations of high levels of conduct problems, callous-unemotional traits, and psychopathic traits, and one comparison group, by gender, with control for age, and parents’ SES and origin, M (SD) or %

	CP only^a^		CU only^b^		PP only^c^		CPCU^d^		CPPP^e^		Low on all^f^		
Boys	(*n =* 28)		(*n =* 45)		(*n =* 23)		(*n =* 14)		(*n =* 73)		(*n* = 499)		*F(* ${\eta}_p^2$ *)*
GD	1.11	(.03)^ce^	1.09	(.03)^ce^	2.00	(.04)^abdef^	1.15	(.05)^ce^	2.16	(.02)^abcdf^	1.04	(.01)^ce^	578.75***(.81)
CU	1.40	(.06)^bcdef^	2.23	(.05)^aef^	2.20	(.06)^aef^	2.30	(.08)^aef^	2.62	(.04)^abcdf^	1.19	(.01)^abcde^	395.63***(.75)
INS	1.63	(.06)^cef^	1.58	(.05)^cef^	2.48	(.07)^abdef^	1.85	(.09)^cef^	2.96	(.04)^abcdf^	1.41	(.01)^abcde^	323.68***(.71)
CP	2.49	(.07)^bcef^	2.01	(.08)^cdef^	2.03	(.08)^abdef^	2.66	(.10)^bcef^	3.26	(.05)^abcdf^	1.39	(.02)^acde^	346.99***(.72)
Fearlessness	1.36	(.08)^ce^	1.54	(.06)^cef^	2.11	(.09)^abdf^	1.59	(.11)^cf^	2.39	(.05)^abcf^	1.24	(.02)^bcde^	105.42***(.44)
ADHD	2.14	(.10)^cef^	2.01	(.08)^cdef^	2.62	(.11)^abef^	2.49	(.14)^bef^	3.36	(.06)^abcdf^	1.70	(.02)^abcde^	146.28***(.52)
													Pearson *χ*^2^
Stable Fearlessness	3.4^cde^		12.^ef^		12.0^abf^		21.4^f^		41.5^abf^		1.5^bcde^		171.37***
Stable ADHD	0.0^e^		6.5^e^		20.0^f^		7.1		48.8^abf^		1.7^ce^		224.62***
Stable CP + ADHD	0.0^e^		0.0^e^		4.0^e^		7.1		34.1^abcf^		0.7^e^		183.97***
Girls	(*n =* 35)		(*n =* 31)		(*n =* 17)		(*n =* 11)		(*n =* 69)		(*n* = 486)		*F(* ${\eta}_p^2$ *)*
GD	1.12	(.03)^ce^	1.11	(.03)^ce^	1.86	(.04)^abdef^	1.14	(.05)^ce^	2.10	(.02)^abcdf^	1.04	(.01)^ce^	617.55***(.83)
CU	1.18	(.04)^bcde^	2.03	(.05)^aef^	2.08	(.06)^aef^	2.13	(.08)^af^	2.35	(.03)^abcf^	1.11	(.01)^bcde^	395.71***(.76)
INS	1.54	(.05)^cef^	1.51	(.05)^cef^	2.22	(.07)^abdef^	1.61	(.09)^cef^	2.64	(.04)^abcdf^	1.34	(.01)^abcde^	251.77***(.66)
CP	2.09	(.06)^bcef^	1.28	(.06)^ade^	1.53	(.08)^adef^	2.37	(.10)^bcef^	2.71	(.04)^abcdf^	1.26	(.02)^acde^	290.20***(.69)
Fearlessness	1.32	(.06)^ce^	1.73	(.08)^ce^	1.95	(.09)^abdf^	1.32	(.11)^ce^	2.14	(.04)^abdf^	1.21	(.02)^ce^	91.04***(.42)
ADHD	1.97	(.08)^ef^	1.73	(.08)^ce^	2.25	(.11)^bef^	2.03	(.14)^ef^	2.85	(.06)^abcdf^	1.49	(.02)^acde^	113.66***(.47)
													Pearson *χ*^2^
Stable fearlessness	0.0		3.1^e^		15.8^f^		0.0		34.2^abf^		0.4^ce^		179.16***
Stable ADHD	5.3		3.1^e^		21.1^f^		9.1		42.1^abf^		1.4^ce^		180.18***
Stable CP + ADHD	5.3		0.0^e^		0.0		0.0		28.9^bf^		0.6^ae^		144.54***

CP only = Above 0.5 *SD* (within gender) rating of conduct problems; CU only = Above 0.5 *SD* (within gender) rating of CU traits; PP only = Above 0.5 *SD* (within gender) rating of psychopathic personality (GD + CU + INS); CPCU = Above 0.5 *SD* (within gender) rating of conduct problems and CU traits; CPPP = Above 0.5 *SD* (within gender) rating of conduct problems and psychopathic traits; Low on all = Below 0.5 *SD* (within gender) ratings of all psychopathic traits and CP

Levene’s test indicated non-equal variance. Superscripts indicate significant differences between groups, i.e., the letter a indicates that the group (column) differs significantly from group a (CP only) in levels of the variable in question (row), b from group b (CU only) and so on. Group means in the ANCOVA were Bonferroni corrected to adjust for multiple comparisons

*p < .05 **p < .01 ***p < .001

### Predicting Fearlessness, ADHD Symptoms, and Combined CP and ADHD Symptoms

To test the hypothesis that the combination of CP and psychopathic personality was a stronger predictor of stable fearlessness, stable ADHD symptoms, and a stable combination of CP + ADHD symptoms, logistic regressions were implemented to test predictive ability of the identified groups (results are presented in Table 3).

**Table 3:** Model evaluation and odds ratios from logistic regression analyses, for boys and girls respectively, of the predictive ability of combinations of CP and psychopathic personality traits for stable ADHD, stable fearlessness, and combination of stable CP and ADHD over a 2-year follow-up

Boys	Stable Fearlessness					Stable ADHD					Stable CP + ADHD				
	OR	*SE* (95% CI)	Wald’s χ^2^	*df*	*p*	OR	*SE* (95% CI)	Wald’s χ^2^	*df*	*p*	OR	*SE* (95% CI)	Wald’s χ^2^	*df*	*p*
Constant	.09***	.60	16.51	1	.00	.11***	.55	17.71	1	.00	.06***	.66	17.34	1	.00
Age	.99	.01 (.97,1.02)	.64	1	.42	1.00	.01 (9.73,1.02)	.21	1	.65	.99	.01 (.97,1.02)	.21	1	.64
SES	.63*	.19 (.43,.91)	6.18	1	.01	.07*	.16 (.51,.96)	4.87	1	.03	.71	.21 (.47,1.06)	2.85	1	.09
Origin	.88	.34 (.46,1.71)	.14	1	.71	1.61	.26 (.95,2.73)	3.16	1	.08	1.51	.33 (.79,2.90)	1.52	1	.22
CP only	.57	1.04 (.07,4.34)	.30	1	.59	+∞1		.001	1	1.00	+∞1		.001	1	1.00
CU only	2.69*	.47 (1.06,6.80)	4.36	1	.04	.75	.62 (.23,2.51)	.22	1	.64	+∞1		.001	1	1.00
PP only	2.67	.66 (.74,9.64)	2.26	1	.13	2.77	.53 (.97,7.89)	3.64	1	.06	.80	1.04 (.10,6.16)	.05	1	.83
CPCU	4.68*	.68 (1.24,17.73)	5.16	1	.02	.84	1.05 (.11,6.58)	.03	1	.87	1.47	1.06 (.19,11.60)	.13	1	.72
CPPP	11.27***	.30 (6.30,20.19)	66.44	1	.00	10.96***	.27 (6.4,18.73)	76.60	1	.00	10.33***	.30 (5.73,18.63)	60.24	1	.00
χ^2^	76.88***					98.58***					75.10***				
Nagelkerke R^2^	.18					.20					.20				
Model	χ^2^	*df*		*p*		χ^2^	*df*		*p*		χ^2^	*df*			*p*
Likelihood ratio	76.88	8		.00		98.58	8		.00		75.10	8			.00
Wald	397.68	1		.00		382.09	1		.00		396.69	1			.00
Goodness-of-fit^1^	4.39	8		.82		13.39	8		.10		11.12	8			.20
Girls	Stable Fearlessness					Stable ADHD					Stable CP + ADHD				
	OR	*SE* (95% CI)	Wald’s χ^2^	*df*	*p*	OR	*SE* (95% CI)	Wald’s χ^2^	*df*	*p*	OR	*SE* (95% CI)	Wald’s χ^2^	*df*	*p*
Constant	.04***	.84	15.32	1	.00	.09***	.61	15.05	1	.00	.03***	.86	15.80	1	.00
Age	.99	.02 (.95,1.02)	.58	1	.45	.99	.01 (.97,1.02)	.53	1	.47	.99	.02 (.96,1.03)	.15	1	.70
SES	.80	.27 (.48,1.35)	.71	1	.40	.73	.20 (.50,1.07)	2.62	1	.11	.65	.27 (.38,1.09)	2.70	1	.10
Origin	1.32	.42 (.58,3.03)	.44	1	.51	1.08	.34 (.59,2.08)	.05	1	.82	1.15	.44 (.48,2.73)	.09	1	.76
CP only	+∞1		.001	1	1.00	.96	.75 (.22,.4.20)	.001	1	.96	2.52	.78 (.54,11.71)	1.40	1	.24
CU only	1.38	1.06 (.17,10.93)	.09	1	.76	.47	1.03 (.06,3.56)	.53	1	.47	+∞1		.001	1	1.00
PP only	6.50*	.80 (1.35,31.39)	5.43	1	.02	3.60	.66 (.99,13.12)	3.77	1	.05	+∞1		.001	1	1.00
CPCU	+∞1		.001	1	1.00	1.50	1.07 (.19,12.14)	.15	1	.70	+∞1		.001	1	1.00
CPPP	24.83***	.37 (11.97,51.51)	74.40	1	.00	11.72***	.30 (6.54,20.98)	68.48	1	.00	15.30***	.37 (7.38,31.74)	53.75	1	.00
χ^2^	87.07***					73.35***					62.46***				
Nagelkerke R^2^	.30					.19					.23				
Model	χ^2^	*df*	*p*			χ^2^	*df*	*p*			χ^2^	*df*		*p*	
Likelihood ratio	87.07	8	.00			73.35	8	.00			62.46	8		.00	
Wald	342.75	1	.00			363.53	1	.00			334.44	1		.00	
Goodness-of-fit^1^	4.65	8	.79			3.81	8	.19			8.30	8		.41	

OR = Odds ratio; *SE* = Standard Error; Above 0.5 *SD* (within gender) rating of conduct problems; CU only = Above 0.5 *SD* (within gender) rating of CU traits; PP only = Above 0.5 *SD* (within gender) rating of psychopathic personality (GD + CU + INS); CPCU = Above 0.5 *SD* (within gender) rating of conduct problems and CU traits; CPPP = Above 0.5 *SD* (within gender) rating of conduct problems and psychopathic traits. Odds ratios represent the expected increase in dependent variables with a one-unit increase of the independent variable in question

^a^ Hosmer-Lemeshow

*p < .05 ***p < .001

The groups, i.e., CP only, CU only, PP only, CPCU, and CPPP, were entered into the analyses as dummy independent variables, to determine which combinations of CP and psychopathic traits were the stronger predictors of stable fearlessness, stable ADHD symptoms and stable CP + ADHD symptoms. Results from the regression analyses, presented in Table 3, show that, as expected, the combination of CP and the full psychopathic personality (CPPP) clearly is the stronger predictor of both stable fearlessness, ADHD symptoms, and CP + ADHD symptoms for both boys and girls. CU only was a significant, but weak, predictor of stable fearlessness among boys, but not among girls, whereas PP only was a strong and significant predictor of stable fearlessness among girls, but non-significant among boys. CP only was not a significant predictor of any of the outcome variables for neither boys nor girls. However, in some cases, the models failed to converge due to quasi-complete separation, as there were no children displaying high levels of the dependent variable in question, in some groups. Nevertheless, likelihood ratio tests are still valid, which in this case, together with other model fit indices (i.e., Wald’s statistic and Hosmer-Lemeshow), indicate good model fits for all models tested. We also tested the models without the problem predictors (not reported in the table, but can be provided upon request), with similar results and conclusions as those reported, only with slightly higher odds ratios.

## Discussion

The aim of this study was to compare children with conduct problems (CP) only, children with CP and callous-unemotional (CU) traits, and children with CP and psychopathic personality on levels of fearlessness and ADHD symptoms, both cross-sectionally and developmentally. In line with our hypotheses, the analyses showed that children with combined high levels of CP and psychopathic personality displayed higher levels of both fearlessness and ADHD symptoms than other children. Most importantly, they displayed higher levels than children with CP and concurrent CU traits only, both at baseline, at the 2-year follow-up, and of stable levels of fearlessness and ADHD symptoms over time. Children with CP and psychopathic personality also displayed higher levels of a stable combination of CP and ADHD symptoms over the two-year follow-up. This was true for boys as well as girls. Thus, the results discovered in this study show that children with CP and psychopathic personality exhibit higher levels of traits and behaviors that increase the risk for severe and persistent CP than children with high levels of CP and CU traits.

Fearlessness has in previous research been related primarily to children’s levels of CU traits (Barry et al. 2000; Frick and Sheffield Morris 2004; Pardini 2006). In the present study, however, children with high levels of CP and CU traits were not worst off when it came to fearlessness, neither cross-sectionally, nor developmentally over two years. Rather, children with high levels of CP and psychopathic personality displayed the highest levels of fearlessness in this study. Even though the differences in fearlessness at baseline were rather small, the CPPP group showed a higher prevalence of stable fearlessness than the CPCU children. This suggests that over and above CU traits, the other dimensions of psychopathic traits, i.e., grandiosity and deceitfulness, and impulsivity and need for stimulation, are of importance for the children’s levels of fearlessness over time. Thus, they are of importance for the children’s maintained levels of risk for severe CP in the future. Our analytical approach also made it possible to compare children with CP only, CU only, and PP only to children with CP and concurrent psychopathic traits. The results showed that while CU traits was a (weak) significant predictor of fearlessness among boys, and PP a significant predictor of fearlessness among girls, there seems to be something in the combination of early-onset CP and concurrent psychopathic traits (primarily all three dimensions) that is strongly associated to the development and maintenance of fearlessness, ADHD symptoms, and combined CP and ADHD symptoms over time. These results are in line with previous results from the same cohort (Frogner et al. 2016), showing that it is the combination of early-onset CP and psychopathic personality that best predicts future CP.

Previous findings often suggest that ADHD symptoms are related to childhood CP, adult criminal behavior, and to high scores on all three dimensions of psychopathic traits rather than to separate dimensions alone (DeLisi et al. 2014; Frick et al. 2000). Also, we investigated the combination of CP and ADHD symptoms, as this combination is related to a greater risk for persistent antisocial behavior. In the present study, children with CP and psychopathic personality displayed the highest levels of both baseline and stable ADHD symptoms, and ADHD symptoms in combination with CP. This provides further support for the idea that children with CP in combination with the full psychopathic personality should be considered an important subgroup of children with CP when it comes to risk and severity.

All analyses were conducted for boys and girls separately in order to possibly identify gender specific patterns, rather than to test for gender differences within analyses. Results were quite similar, showing that fearlessness and ADHD symptoms were prominent risk factors in relation to CP and psychopathic personality among both genders. Also, the CPPP groups showed the strongest relation to stable high levels of fearlessness, ADHD symptoms, and the combination of CP and ADHD symptoms among both boys and girls. Previous research on fearlessness and ADHD symptoms, both alone and in combination with CP and psychopathic traits, have shown mixed results among boys and girls. The results from this study showed similar patterns in levels of fearlessness and ADHD symptoms among boys and girls both at baseline and developmentally, and similar associations between risk factors, CP, and psychopathic traits appeared. This indicates that while commonly displaying early-onset CP to different extents, both boys and girls with CPPP displayed substantial levels of fearlessness, ADHD symptoms, and CP + ADHD symptoms in early childhood.

### Diagnostic Practice and Treatment

In an extensive review, Frick et al. (2014) assess existing studies on CU traits in relation to CP among children, based on their relevance for the *DSM-5* LPE specifier. None of the highest rated (i.e., most relevant) studies on the relation between CU traits and CP control for other dimensions of psychopathic traits (see Kahn et al. 2012; McMahon et al. 2010; Pardini et al. 2012). We believe that this is problematic, if the aim is to achieve knowledge that can result in more effective diagnostics and interventions. Results from a recent study, based on the same sample as the one used in the present study, showed that the combination of CP and psychopathic personality including all three dimensions of psychopathic traits, was a stronger predictor of stable CP over a 2-year follow-up than the combination CP and CU traits among boys (Frogner et al. 2016). Also, the development of other psychopathic traits seems to follow the development of CP and CU over time (e.g., Klingzell et al. 2016), and studies have shown that the predictive ability of CU traits for CP is reduced when controlling for other psychopathic traits (Barry et al. 2000; Corrado et al. 2004; Frick et al. 2000). It is also notable that the group sizes in the present study, which features a fairly sizeable (non-referred) sample, suggest, that children with CP with concurrent CU traits only and not the other psychopathic personality dimensions, are hard to identify. Few children displayed a combination of CP and CU traits when the children with psychopathic personality – that is, children who scored high on all three dimensions of psychopathic personality – were separated from those with CU only. The children with CP and psychopathic personality make up clearly larger groups in the sample among both boys and girls, than children with CP and CU traits only.

By using fearlessness and ADHD symptoms, two theoretically relevant and empirically established risk factors for CP, as examples, this study has shown that children with a combination of CP and the full psychopathic personality display higher levels of substantial risk factors for CP than children with CP and concurrent CU traits only. Furthermore, based on the results from this study, it is important to note that early-onset CP *without* concurrent psychopathic traits did not significantly predict stable levels of neither fearlessness nor ADHD symptoms. This is information that can aid the identification of children at risk at an early age, as there seems to be something in the combination of CP and psychopathic traits, or rather psychopathic personality, that is related to prevalence of grave risk for the future for children with CP.

Both fearlessness and ADHD symptoms also imply specific treatment needs. For example, children with high levels of fearlessness are commonly insensitive to punishment and negative consequences, which demands interventions applying alternative ways of socialization (Dadds and Salmon 2003; Frick and Sheffield Morris 2004; Shaw et al. 2003). Similarly, children with high ADHD symptoms do, according to research, respond well to stimulant medication, which have been shown to decrease both ADHD symptoms and aggressive behavior (Blader et al. 2013). Knowledge like this make these risk factors important to study among subgroups of children with CP in order to render more informed diagnoses, and thus increasing possibilities for better tailored interventions.

More research is needed on children with CP in combination with psychopathic personality, particularly in comparison to those with the CPCU combination. We need to take into account possible genetic, familial, and environmental influences as well. This way, additional knowledge can be generated on useful ways to subgroup children with CP, and whether this should have implications for the way the CD diagnosis in general, and the LPE specifier in particular, is implemented. Consequently, there are compelling arguments that the CD LPE specifier is in need of more scrutiny. Based on our results, together with a growing body of research investigating children with CP and psychopathic personality, these are the children that are likely to display CP at later points in life. Since previous research has not controlled for levels of other psychopathic traits in children with CP and CU traits to a satisfying extent, these comparisons and controls need more attention in research to build an adequate knowledge base for the revision of the DSM and the ICD (see Salekin 2016).

### Limitations and Strengths

The current study had several limitations. Some of the models in the logistic regression analyses suffered from non-convergence due to quasi-complete separation, i.e., some groups of children did not display certain traits or behaviors tested, resulting in a cell count of zero. However, likelihood ratios are still valid for these models, as are maximum likelihood estimates for other variables in the models, which indicates an acceptable model fit despite these issues. Disregarding the statistical issues, it is an indication in itself that, for example, no girls with high levels of CPCU displayed stable levels of fearlessness or the CP + ADHD symptoms combination in this sample (using our cutoffs), while girls with high CPPP displayed notable odds ratios for stable levels of fearlessness and CP + ADHD symptoms.

Furthermore, due to the dichotomization of the independent variables, i.e., the grouping variables, the groups of children were in some cases rather small, rendering statistical power issues, which might have increased the risk of false negatives. This could in turn contribute to not finding existing differences where they might actually occur. The tendency in the analyses was that CPPP children displayed higher levels of stable fearlessness, ADHD symptoms, and CP + ADHD symptoms than all the other groups, with percentage differences between groups of up to over 40%. The percentage differences between the CPPP and CPCU groups were so substantial, that with larger groups the differences would most likely have been statistically significant. However, the results in this study indicate that children with CPPP are more likely to manifest these traits and behaviors consistently over time.

The study relies on teacher ratings only, which might limit the possibilities of generalizing the results. Another possible consequence of relying on single rater information for both independent and dependent variables is that this could yield regression coefficient estimates that were positively inflated by shared method variance. On the other hand, using teacher ratings can also be considered a strength, as most children spend a lot of their waking hours with teachers, and teachers therefore get to observe children in various situations and interactions with peers and adults (Abikoff et al. 1993). Furthermore, teachers may be better able than parents to rate children’s normative and non-normative behaviors, thanks to their education and experience. For this reason, the CPTI was designed to be used by teachers, and as we wanted to avoid that results might be influenced by rater variance by using different raters for different variables, we used teacher ratings only for CP as well in this study.

The prospective longitudinal design, and the large representative sample are substantial strengths of the study, as well as the low attrition, which adds to the validity of the results. Also, albeit being a rather new assessment tool, the CPTI has so far proved good reliability and validity in several (European) samples (Colins et al. 2014; Colins et al. 2016a, 2016b; Somma et al. 2015).

### Future Research and Practical Implications

This study highlights children with psychopathic personality as an important subgroup of children with CP, with levels of risk traits and behaviors that undoubtedly bring aggravating circumstances to their behavioral development. Thus, this group of children should not be neglected in diagnostic practice as a consequence of the focus on children displaying CP and concurrent CU traits only. To improve diagnostic practice, predictive accuracy, and treatment responsivity among children with CP, more knowledge is needed pertaining to what risky traits and behaviors to look for, at what point in life to start looking for them, what risks the traits and behaviors bring for the future, and what treatments ensure the most optimal outcomes given specific risk factors. Studies that compare children with concurrent CP and CU traits to children with CP and the full psychopathic personality on different aspects relevant for diagnostic practice and treatment, such as prevalence of risk factors, are still lacking. There is also a need to test whether these results hold in referred samples. To implement a well-validated CD diagnosis there is a need to capture a range of psychopathic traits over and above CU traits, to identify children at risk for severe and persistent CP.

## Footnotes

1 In Sweden, the year a child turns six years of age he/she transfers from *preschool* to *preschool class,* which is a part of the primary school system. During the fall of 2011, children born in 2005 advanced to preschool class, and in 2012, children born in 2006 did the same, leaving only children born in 2007 in preschool for the 2-year follow up data collection.
